# Metal-free double azide addition to strained alkynes of an octadehydrodibenzo[12]annulene derivative with electron-withdrawing substituents

**DOI:** 10.3762/bjoc.20.191

**Published:** 2024-09-04

**Authors:** Naoki Takeda, Shuichi Akasaka, Susumu Kawauchi, Tsuyoshi Michinobu

**Affiliations:** 1 Department of Materials Science and Engineering, Tokyo Institute of Technology, 2-12-1 Ookayama, Meguro-ku, Tokyo 152-8552, Japan

**Keywords:** annulene, click chemistry, polymerization, strain-promoted azide–alkyne cycloaddition

## Abstract

Strain-promoted azide–alkyne cycloaddition (SpAAC) is a powerful tool in the field of bioconjugation and materials research. We previously reported a regioselective double addition of organic azides to octadehydrodibenzo[12]annulene derivatives with electron-rich alkyloxy substituents. In order to increase the reaction rate, electron-withdrawing substituents were introduced into octadehydrodibenzo[12]annulene. In this report, the synthesis of new octadehydrodibenzo[12]annulene derivatives, regioselective double addition of organic azides, and an application to crosslinking polymers are described.

## Introduction

The strain-promoted azide–alkyne cycloaddition (SpAAC) is one of the most representative metal-free click chemistry reactions [1–5]. SpAAC has been mainly employed in bioconjugation in the fields of chemical biology and medicinal chemistry due to its high efficiency under physiologically active conditions and the absence of any toxic metal ions. SpAAC is biorthogonal, which allows for the specific labeling and imaging of biomolecules even in living cells and organisms. Recently, a more rapid click reaction was desired and the strain-promoted oxidation-controlled cyclooctyne-1,2-quinone cycloaddition (SPOCQ) was developed and employed in the same fields of chemical biology [6–7]. On the other hand, the use of the SpAAC in materials science was slow. We developed another class of metal-free click chemistry reactions, such as the [2 + 2] cycloaddition–retroelectrocyclization (CA-RE) between electron-rich alkynes and electron-deficient olefins [8]. The [2 + 2] CA-RE click reactions were employed to produce a variety of functional materials, such as nonlinear optical chromophores [9–10], super acceptors [11–12], ion sensing D–A systems [13], and crosslinked polymers [14–17]. Since crosslinking polymers requires high reaction efficiency under mild conditions, developing such reactions is crucial.

**Figure 1:**
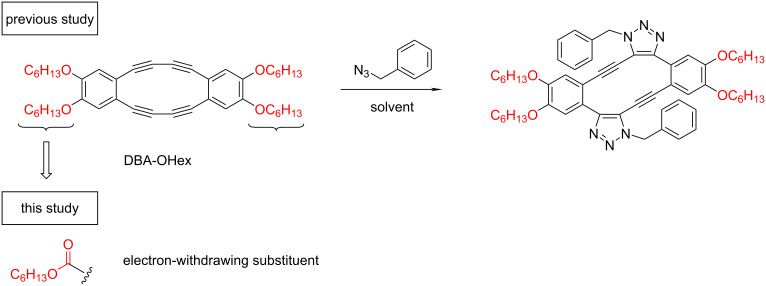
Previously reported regioselective double azide addition to DBA with hexyloxy substituents and molecular design in this study.

We previously reported the regioselective double azide addition to octadehydrodibenzo[12]annulene with hexyloxy substituents (DBA-OHex), which are readily accessible by the oxidative acetylenic coupling of a 1,2-diethynylbenzene derivative (Figure 1) [18]. The chemical stability of DBAs depends on the electronic character of the substituents. Electron-donating alkyloxy groups are known to enhance the stability of DBAs. On the other hand, DBAs substituted with electron-withdrawing groups have been little studied [19], and their chemical stability and physical properties are not well understood. In this paper, a new DBA substituted with ester groups was synthesized, and the double azide addition was comprehensively investigated. Finally, the double azide addition reaction was applied to polymer crosslinking and the mechanical properties of the self-standing polymer films were compared.

## Results and Discussion

### Strain-promoted azide–alkyne cycloaddition

**Scheme 1:**
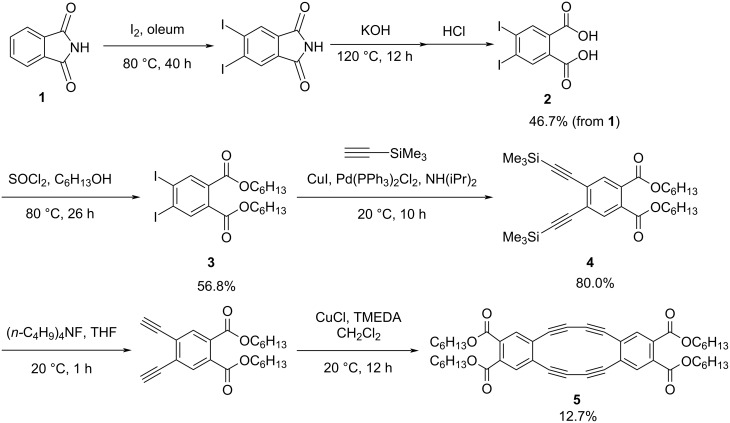
Synthesis of DBA **5**.

Octadehydrodibenzo[12]annulene (DBA) with electron-withdrawing carbonyl substituents **5** was prepared from phthalimide (**1**, Scheme 1). Iodination followed by hydrolysis afforded 4,5-diiodophthalic acid (**2**) in 46.7% yield. Esterification with 1-hexanol yielded compound **3** in 56.8% yield and the subsequent Sonogashira coupling with trimethylsilylacetylene provided compound **4** in 80.0%. Silyl deprotection with (*n-*C_4_H_9_)_4_NF in THF followed by acetylenic oxidative dimerization under Hay conditions produced the desired DBA **5** in 12.7% yield. It should be noted that a thermodynamically more stable trimeric macrocycle was also formed in 17.7%, but it was not isolated and purified because it was outside the scope of this study.

**Figure 2:**
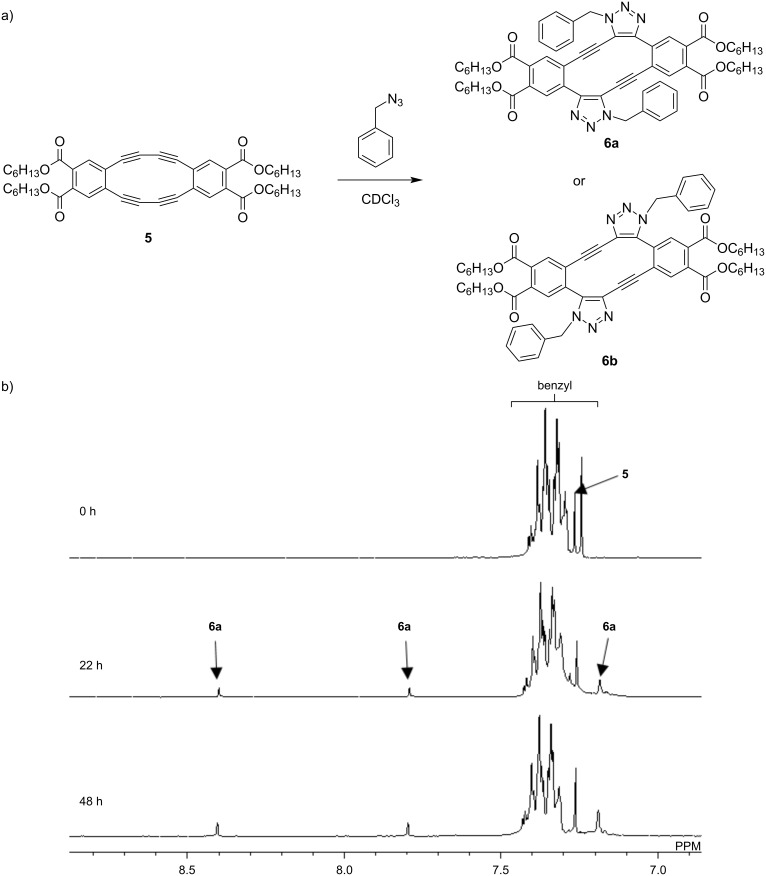
(a) Strain-promoted azide–alkyne cycloaddition between DBA **5** and benzyl azide and (b) ^1^H NMR spectral change at 30 °C in CDCl_3_.

Next, DBA **5** was subjected to the SpAAC with benzyl azide. When two equivalents of benzyl azide were added to a solution of **5** in CDCl_3_ at a controlled temperature of 30 °C, the reaction slowly proceeded (Figure 2). After 22 h, the original peak ascribed to starting compound **5** decreased, while a set of new peaks appeared at 8.40 ppm, 7.79 ppm, and 7.20–7.18 ppm. Considering the high symmetry, no monoadducts were formed and the regioselective double azide addition occurred. In other words, 1,4-disubstituted-5-ethynyltriazole derivative (in-) (**6a**) or 1,5-disubstituted-4-ethynyltriazole derivative (out-) (**6b**) adducts were formed. To determine the chemical structure of the product, the NOESY measurement was conducted. The NOESY spectrum suggested the intermolecular through-space coupling between two benzene protons (Figure S10 in Supporting Information File 1). This result indicates that the formed double azide adduct is **6a**, which is consistent with our previous report [18].

**Figure 3:**
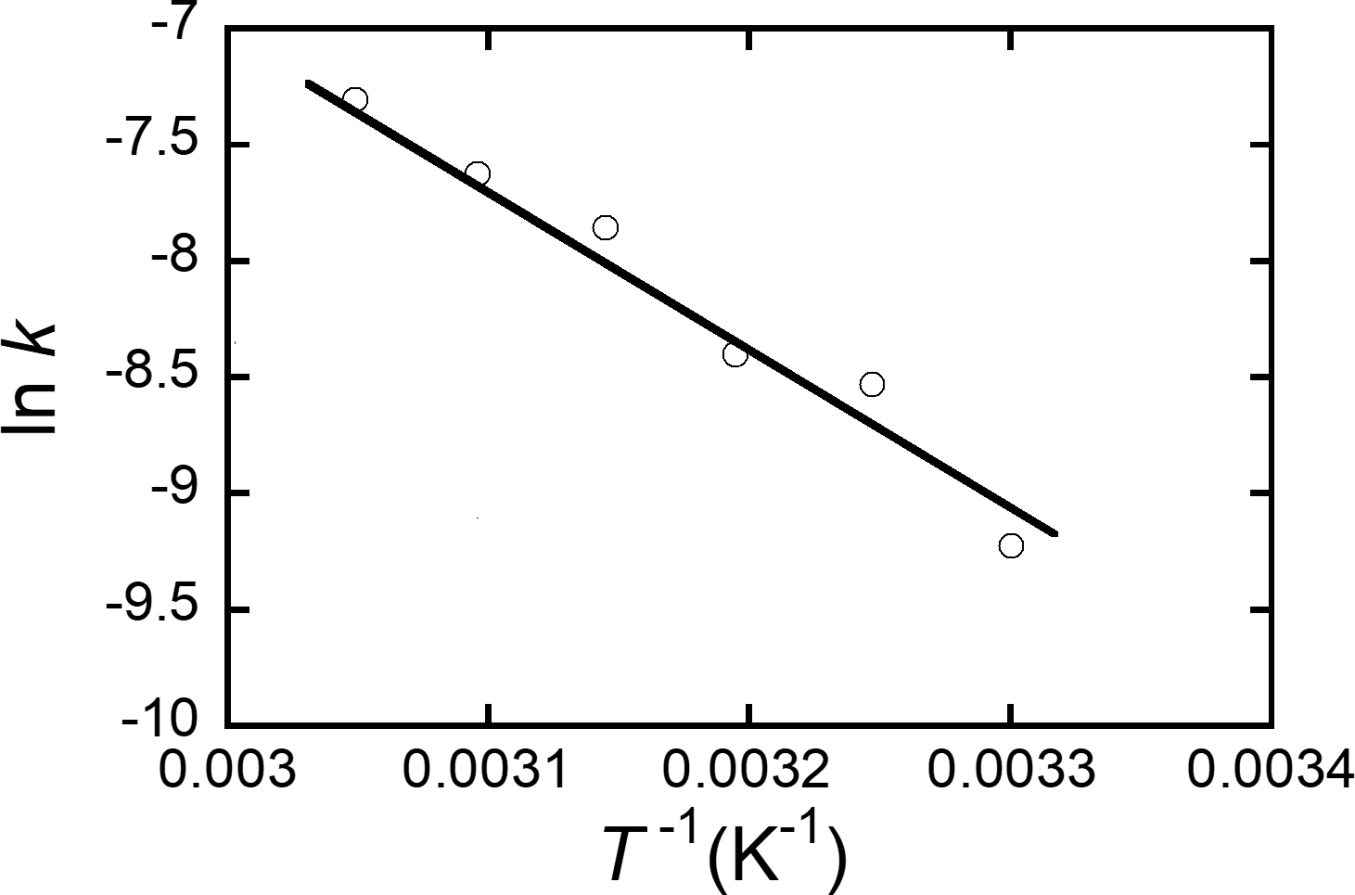
Arrhenius plots of the rate constants for the reaction between **5** and benzyl azide in CDCl_3_.

The double azide addition was further investigated by changing the reaction temperature. The rate constant was determined by the temperature-dependent ^1^H NMR spectra in CDCl_3_. The reaction kinetics followed a second-order reaction. Since no monoadducts were formed, the rate-determining step is the first azide addition. Based on this fact, the activation energy (*E*_a_) of the reaction between **5** and benzyl azide in CDCl_3_, determined by the Arrhenius plots, was 60.9 kJ mol^−1^ (Figure 3). This value was apparently smaller than those previously reported for the reaction between DBA-OHex and benzyl azide (Table S1 in Supporting Information File 1). However, the *E*_a_ values were previously measured in DMSO-*d*_6_ and C_6_D_6_, and it is known that there is a solvent polarity effect on the reactivity of SpAACs. In order to quantitatively compare the reactivity of DBAs with different substituents, the reaction between DBA-OHex and benzyl azide was also monitored in CDCl_3_. The *E*_a_ in CDCl_3_ was 71.1 kJ mol^−1^ (Table S1 in Supporting Information File 1). This result clearly suggests that DBA **5** with electron-withdrawing substituents has a lower *E*_a_ than DBA with electron-donating substituents.

### DFT calculations

**Figure 4:**
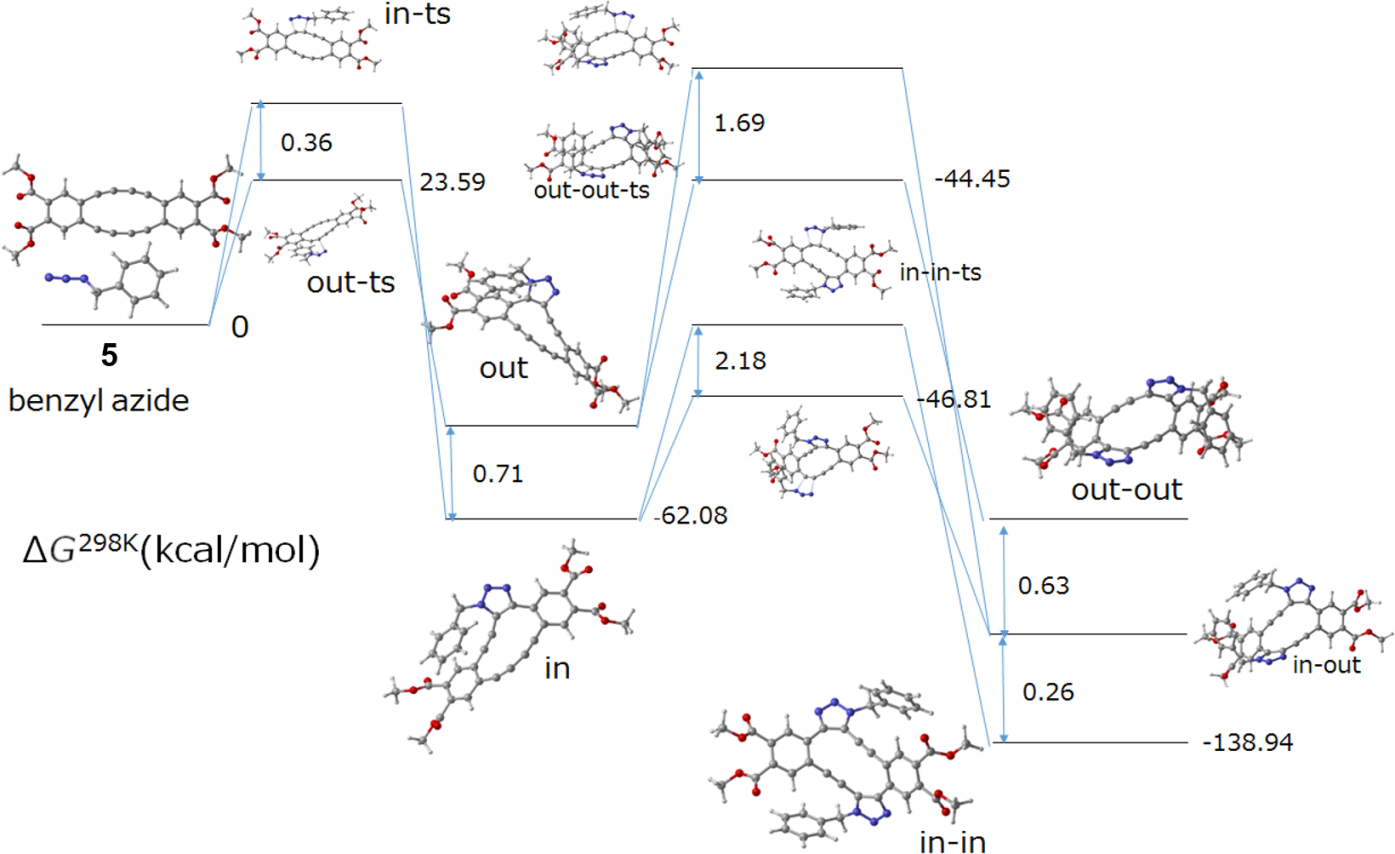
Proposed reaction mechanism for the formation of compound **6a**. Free energy profiles (Δ*G*_298_ in kJ mol^−1^) calculated at the ωB97X-D/6-31G(d,p)/PCM (in CH_2_Cl_2_).

The reaction mechanism was investigated by computational calculations. The reaction mechanism between **5** and benzyl azide was supported by the ωB97X-D/6-31G(d,p) calculations with the CH_2_Cl_2_ polarizable continuum model (PCM) solvent (Figure 4). The reaction initially started with the addition of benzyl azide to one of the internal alkynes of **5**. Although the benzyl group is situated on the interior side of DBA, a more energetically unstable transition state (in-ts) is generated. However, the resulting monoadduct (in) is more energetically demanding than the counter monoadduct (out) due to steric factors. The second azide addition follows this step. The alkyne, which is diagonally positioned relative to the triazole group, shows the highest reactivity due to its significant distortion. This finding correlates with the experimental observation that no monotriazoles were obtained. During the second azide addition, the orientation of benzyl azide is once more controlled. The benzyl group positioned on the inner side results in a thermodynamically less stable transition state (in-in-ts) compared to that on the outer side (in-out-ts), but the thermodynamically stable final product is a regioselective “in-in” adduct, i.e., compound **6a**.

### Optical properties

**Figure 5:**
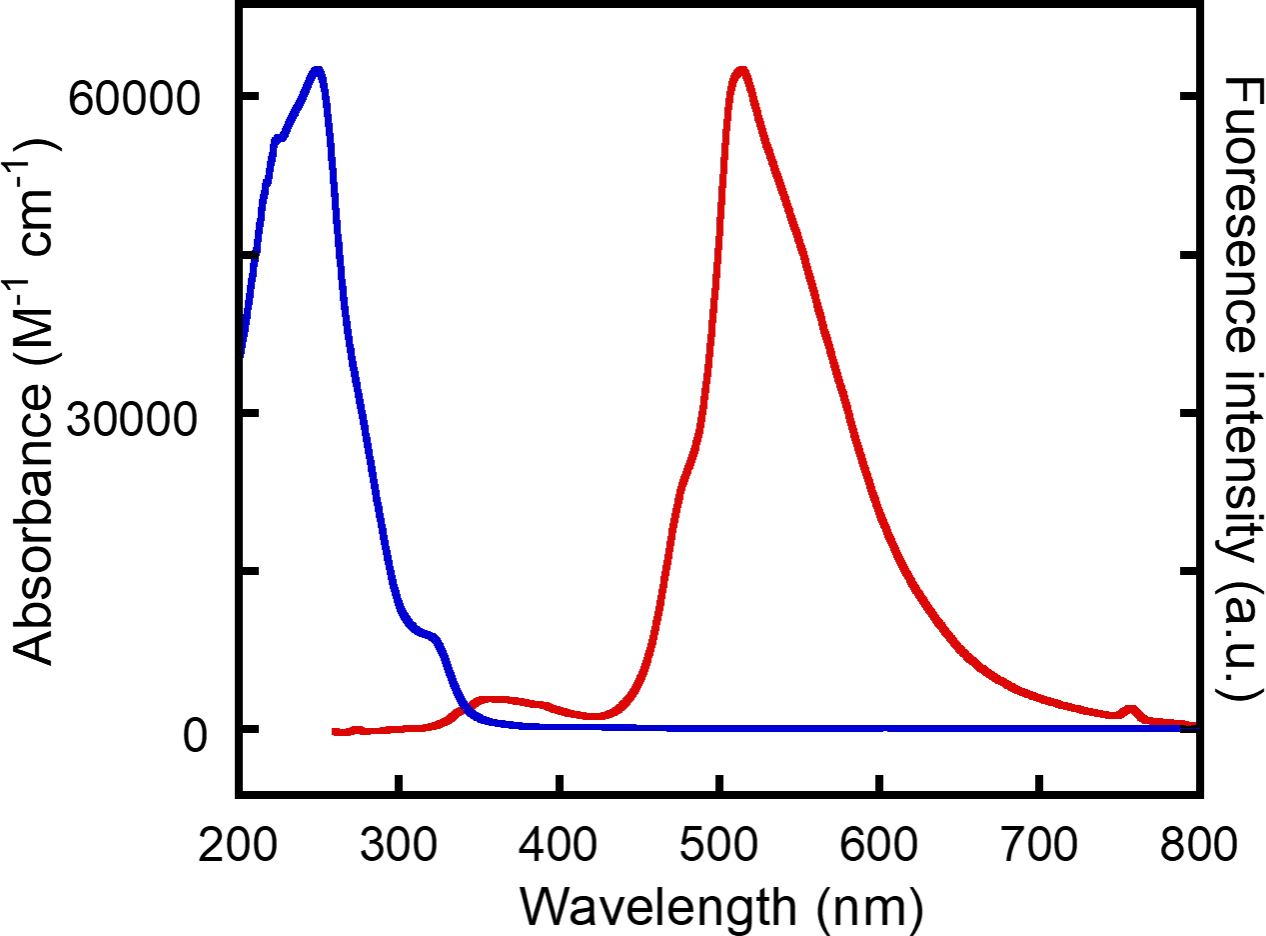
Absorption (blue) and fluorescence (red) spectra of **6a** (2 × 10^−5^ M) in CH_2_Cl_2_.

Absorption and emission spectra of **6a** were measured in CH_2_Cl_2_ (Figure 5). The conjugation is changed and a highly-twisted macrocyle forms by the double azide addition. Thus, compound **6a** shows an ultraviolet absorption peak at 249 nm (λ_max_) and no absorption in the visible region was observed. When excited at 249 nm, an emission band at 513 nm (λ_em_) appeared in the spectrum with a fluorescence quantum yield (Φ) of 7.0%. The absorption and fluorescence spectra were almost independent of solvents. For example, the λ_em_ in CHCl_3_ and THF was 514 nm (Figure S11 in Supporting Information File 1). The double benzyl azide adduct of DBA-OHex displayed similar absorption and emission spectra with a λ_max_ of 247 nm and λ_em_ of 539 nm in CH_2_Cl_2_. Since the Stokes shift of the double benzyl azide adduct of DBA-OHex was 21900 cm^−1^ and larger than that of **6a** (20700 cm^−1^), the Φ was 4.3%. This result suggested that replacing the hexyloxy substituents with electron-withdrawing ones enhanced the fluorescence intensity by approximately 1.6 times.

### Crosslinker application

**Figure 6:**
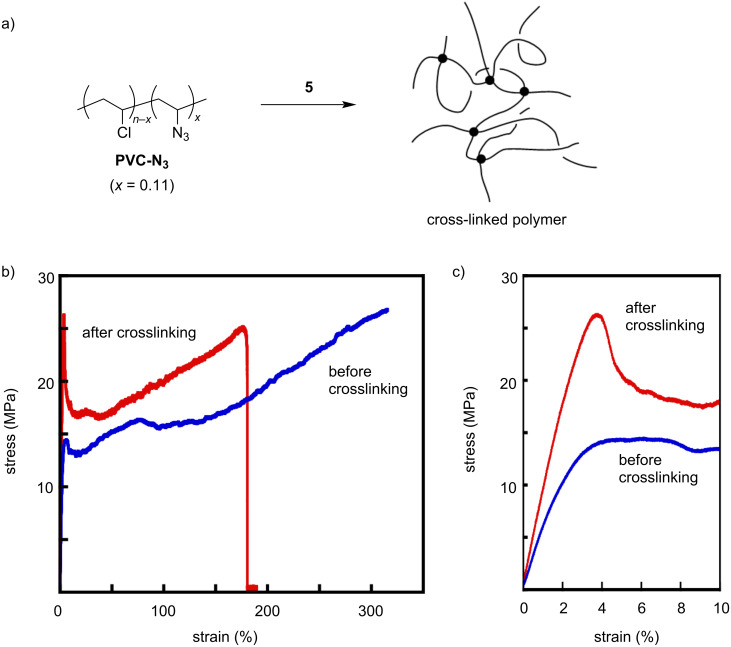
(a) Crosslinking reaction of PVC-N_3_ (*x* = 0.11) with compound **5**. (b,c) Strain-stress curves of PVC-N_3_ before (blue) and after (red) crosslinking.

Using the strain-promoted double azide addition feature, DBA **5** was evaluated as a crosslinker for azidated polymers. After **5** (5 mol %) was added to a partially azidated poly(vinyl chloride) (PVC-N_3_) (*x* = 0.11, *n* = 1000) in THF, the solvent was gradually evaporated on a Teflon boat. The resulting self-standing film became insoluble due to the occurrence of strain-promoted double azide–alkyne cycloaddition (Figure 6a). The mechanical properties of the polymer films were then examined to observe the impact of crosslinking.

A PVC-N_3_ film was prepared by a solvent-cast method on a Teflon boat. The strain-stress (S–S) curve of the PVC-N_3_ film exhibited a breakdown at the strain of >300% with a gradual increase in the stress (Figure 6b). This film did not display any yielding points (Figure 6c). Interestingly, the crosslinking with **5** dramatically changed the mechanical features. The crosslinked film showed a clear yield point at the strain of about 4% with the maximum stress of 26.3 MPa (Table 1). When the applied stress gradually increased, the film displayed a ductility up to the strain of about 180 MPa. Young’s moduli were estimated from the initial slopes of the S–S curves. The Young’s modulus significantly increased from 277 MPa to 777 MPa by crosslinking. This result is consistent with the general understanding of covalent crosslinking of linear polymers. The crosslinking points formed by the SpAAC resulted in a rigid film.

**Table 1:** Mechanical properties of PVA-N_3_ before and after crosslinking.

	Young’s modulus (MPa)	Yield stress (MPa)	Maximum fracture stress (MPa)

PVA-N_3_ (before crosslinking)	277	14.2	26.8
PVA-N_3_ + **5** (after crosslinking)	777	26.3	26.3

## Conclusion

Octadehydrodibenzo[12]annulene substituted with electron-withdrawing groups was successfully synthesized. This molecule underwent SpAAC with two equivalents of benzyl azide under mild conditions to quantitatively form the regioselective symmetric bistriazole product. The electron-withdrawing substituents facilitated the reaction progress as compared to the previously reported electron-donating hexyloxy substituents. The addition pattern was experimentally investigated by 2D NMR, which was also supported by DFT calculations. The bistriazole product displayed fluorescence in the visible range with a fluorescence quantum yield of 7.0%. Finally, the developed metal-free click reaction was employed to crosslink a partially azidated poly(vinyl chloride). The crosslinking proceeded by simply mixing the polymer and crosslinker in THF and evaporating, and the formation of the crosslinked polymer film was confirmed by the strain–stress curves. The developed method is straightforward and has broad applicability, extending to other azidated molecules and polymers.

## Experimental

### Materials

All reagents are purchased from TCI, Aldrich, and Kanto Chemical Co. Inc., and used as received. 4,5-Diiodophthalic acid was prepared according to a literature method [20]. A partially azidated poly(vinyl chloride) (PVA-N_3_) was prepared by stirring poly(vinyl chloride) and NaN_3_ in DMF at room temperature overnight.

### Measurements

NMR spectra were recorded using a JEOL mode Al300 (300 MHz) at room temperature. Deuterated chloroform was used as the solvent unless otherwise stated. Chemical shifts of NMR were reported in ppm relative to the residual solvent peak at 7.26 ppm for ^1^H NMR spectroscopy and 77.6 ppm for ^13^C NMR spectroscopy. Coupling constants (*J*) were given in Hz. The resonance multiplicity was described as s (singlet), t (triplet), and m (multiplet). FTIR spectra were recorded on a JASCO FT/IR-4100 spectrometer in the range from 4000 to 600 cm^−1^. MALDI–TOF mass spectra were measured on a Shimadzu/Kratos AXIMACFR mass spectrometer equipped with a nitrogen laser (λ = 337 nm) and pulsed ion extraction, which was operated at an accelerating potential of 20 kV. THF solutions containing 1 g L^−1^ of a sample, 10 g L^−1^ of dithranol, and 1 g L^−1^ of sodium trifluoroacetate were mixed at a ratio of 1:1:1, and then 1 μL aliquot of this mixture was deposited onto a sample target plate. UV–vis absorption spectra were recorded on a JASCO V-670 spectrophotometer. Fluorescence spectra were recorded on a JASCO FP-8500.

All calculations were carried out using the Gaussian 16 program [21]. The DFT calculations were carried out using the long-range and dispersion-corrected ωB97X-D functional [22]. The 6-31G(d,p) basis set was used for H, C, O, and S atoms [23–24]. The solvent effect of CH_2_Cl_2_ was taken into account by the polarizable continuum model using the integral equation formalism (IEFPCM) [25] for DFT calculations. The optimized molecular structures were verified by vibrational analysis; equilibrium structures did not have imaginary frequencies and transition-state structures had only one imaginary frequency corresponding to the reaction coordinate.

### Synthesis of **6a**

To a solution of **5** in CH_2_Cl_2_, two equivalents of benzyl azide were added and the mixture was stirred at room temperature for 12 h. Evaporation of the solvent quantitatively yielded the target compound. ^1^H NMR (CDCl_3_, 300 MHz, 297 K) δ 8.40 (s, 2H), 7.80 (s, 2H), 7.40–7.18 (m, 10H), 5.53 (s, 4H), 4.34–4.29 (m, 8H), 1.74–1.71 (m, 8H), 1.37–1.32 (m, 24H), 0.92–0.88 (m, 12H); ^13^C NMR (CDCl_3_, 75 MHz, 297 K) δ 166.26, 147.55, 135.76, 133.90, 133.67, 133.36, 131.92, 130.83, 128.74, 128.70, 128.34, 120.99, 119.57, 103.11, 82.18, 66.30, 53.55, 31.39, 28.42, 25.54, 22.48, 13.98; FTIR ν (cm^−1^): 2067 (C≡C), 1730 (C=O); MALDI–TOF MS (dithranol, *m*/*z*): [M]^+^ calcd for C_62_H_70_N_6_O_8_, 1026.53; found, 1026.98.

## Supporting Information

File 1 Experimental section and kinetic study of the reaction of compound **5** and benzyl azide.

## Data Availability

The data that supports the findings of this study is available from the corresponding author upon reasonable request.
