# Supplementary material for: Metal-free double azide addition to strained alkynes of an octadehydrodibenzo[12]annulene derivative with electron-withdrawing substituents
Source: Beilstein J Org Chem. 2024 Sep 4;20:2234–41. doi: 10.3762/bjoc.20.191 (PMC11403804; doi:10.3762/bjoc.20.191)
Supplement: File 1 — Experimental section and kinetic study of the reaction of compound 5 and benzyl azide. [file Beilstein_J_Org_Chem-20-2234-s001.pdf]

BEILSTEIN JOURNAL OF ORGANIC CHEMISTRY

Supporting Information

for

Metal-free double azide addition to strained alkynes of an octadehydrodibenzo[12]annulene derivative with electron-withdrawing substituents

Naoki Takeda, Shuichi Akasaka, Susumu Kawauchi and Tsuyoshi Michinobu

Beilstein J. Org. Chem. **2024,** *20,* 2234–2241. doi:10.3762/bjoc.20.191

Experimental section and kinetic study of the reaction of compound 5 and benzyl azide

License and Terms: This is a supporting information file under the terms of the Creative Commons Attribution License (https://creativecommons.org/licenses/by/4.0). Please note that the reuse, redistribution and reproduction in particular requires that the author(s) and source are credited and that individual graphics may be subject to special legal provisions.
The license is subject to the *Beilstein Journal of Organic Chemistry* terms and conditions: (https://www.beilstein-journals.org/bjoc/terms)

1. Materials

All chemicals were purchased from Tokyo Chemical Industry (TCI), Kanto Chemical Co. Inc., Wako Pure Chemical Industries, and Sigma Aldrich, and used as received unless otherwise stated.

2. Synthesis

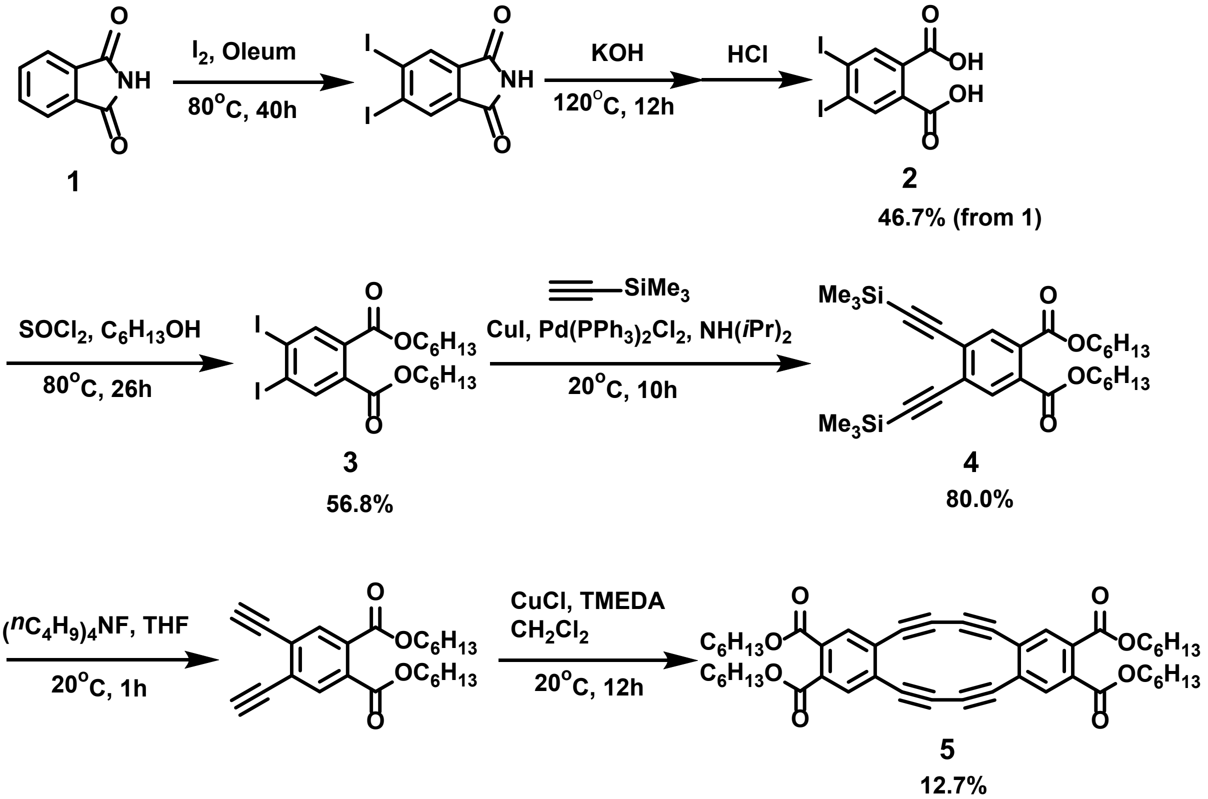

Scheme S1 Synthesis of DBA **5**.

Synthesis of 4,5-diiodophthalic acid (2) [S1]. Into a round-bottle flask, iodine (13.08 g, 51.50 mmol), phthalimide (7.293 g, 50.00 mmol), and fuming sulfuric acid (30 mL) were placed, and the mixture was heated at 80 °C for 40 h. After cooling to room temperature, the yellow precipitate was collected and washed with water, K_2CO_3 aqueous solution, and Na_2SO_3 aqueous solution. The purified yellow solid was dissolved into 10% KOH aqueous solution, and refluxed for 12 h. After cooling to room temperature, conc. HCl solution was added, producing yellow precipitates. The precipitates were dried in vacuum at 60 °C, yielding the desired compound (9.683 g, 46.7%).

^{1}H NMR (DMSO-d_6, 300 MHz, 297 K) δ: 8.32 ppm (2H, s).

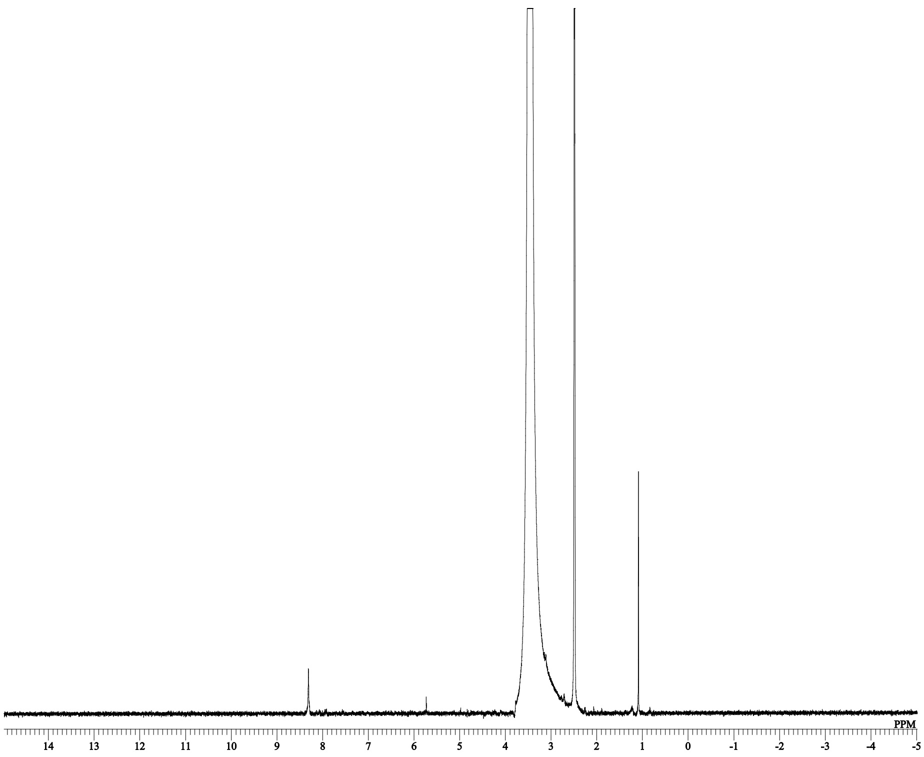

Figure S1 ^{1}H NMR of **2** in DMSO-d_6.

Synthesis of dihexyl 4,5-diiodophthalate (3). Into a 100 mL round-bottle flask, 1-hexanol (25 mL, 0.20 mol) and compound **2** (2.075 g, 4.965 mmol) were placed. After adding $SOCl_2$ (2.5 mL, 35 mmol), the mixture was stirred at 80 °C for 26 h. The mixture was cooled to room temperature and washed with aqueous $NaHCO_3$ solution. The organic layer was extracted with CH_2Cl_2 and dried over Na_2SO_4. Column chromatography afforded the desired compound as pale orange liquid (1.59 g, 56.8%).

^{1}H NMR ($CDCl_3$, 300 MHz, 293 K): δ = 8.14 (2H, s), 4.27 (4H, t, J = 6.8 Hz), 1.72-1.68 (4H, m), 1.33-1.31 (12H, m), 0.90 ppm (6H, t, J = 6.4 Hz). ^{13}C NMR ($CDCL_3$,75 MHz, 297 K) δ: 165.31, 138.93, 132.38, 111.31, 66.07, 31.19, 28.19, 25.34, 22.32, 13.83 ppm. FT-IR (neat): ν = 1919 (C≡C), 1742 (C=O) cm^{-1}. MALDI-TOF MS (dithranol): $[M+Na]^+$ = 609.79.

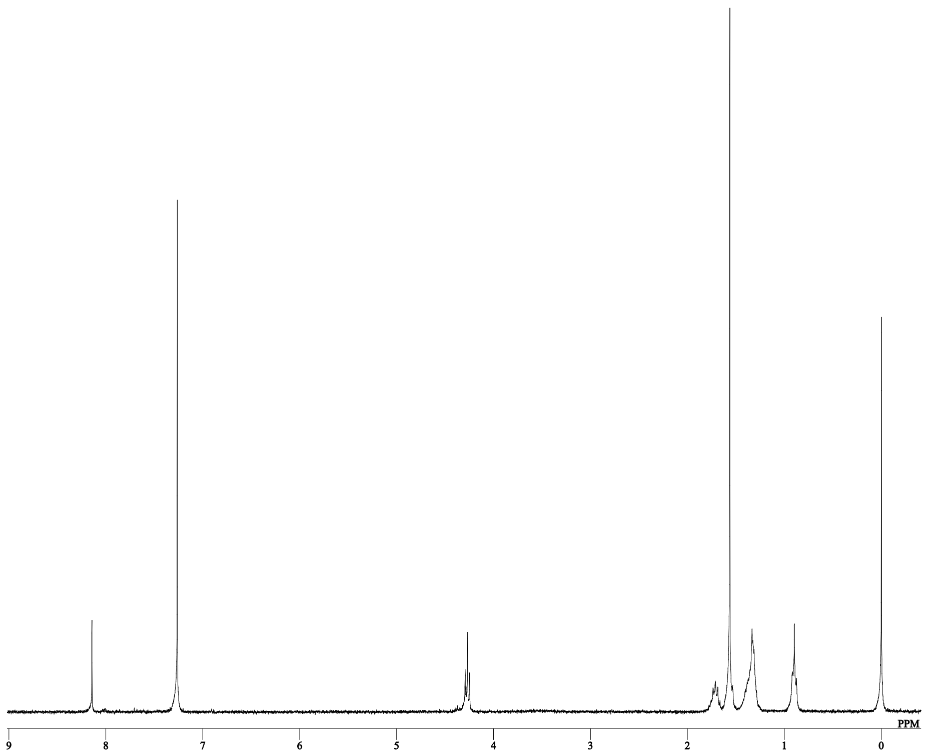

Figure S2 ^{1}H NMR of **3** in $CDCl_3$.

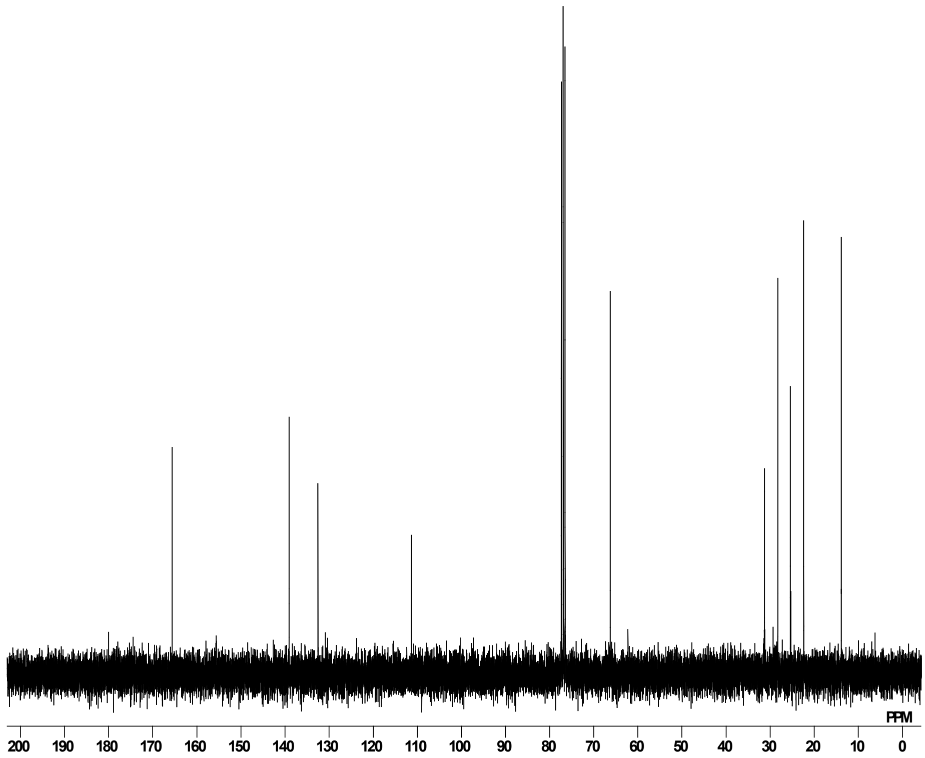

Figure S3 ^{13}C NMR of **3** in $CDCl_3$.

Synthesis of dihexyl 4,5-bis((trimethylsilyl)ethynyl)phthalate (4). Into a 100 mL flask, compound **3** (520.9 mg, 0.8885 mmol), trimethylsilylacetylene (0.60 mL, 4.2 mmol), and diisopropylamine (15 mL) were placed under N_2. $PdCl_2(PPh_3)_2$ (30 mg, 0.043 mmol) and CuI (30 mg, 0.16 mmol) were added and the mixture was stirred at room temperature for 10 h. After filtration, the organic layer was washed with brine, extracted with CH_2Cl_2, and dried over Na_2SO_4. Evaporation and column chromatography afforded the desired compound as orange liquid (376 mg, 80.0%).

^{1}H NMR ($CDCl_3$, 300 MHz, 293 K): δ = 7.77 (2H, s), 4.27 (4H, t, J = 6.8 Hz), 1.81-1.60 (4H, m), 1.44-1.18 (12H, m), 0.91 (6H, t. J = 6.4 Hz), 0.29 ppm (18H, s). ^{13}C NMR ($CDCl_3$, 75 MHz, 297 K) δ: 166.63, 132.90, 131.55, 128.47, 102.37, 101.67, 66.13, 31.62, 28.61, 25.70, 22.59, 13.86, -0.07 ppm. FT-IR (neat): ν = 2067 (C≡C), 1730 (C=O) cm^{-1}. MALDI-TOF MS (dithranol): $[M+Na]^+$ = 549.82.

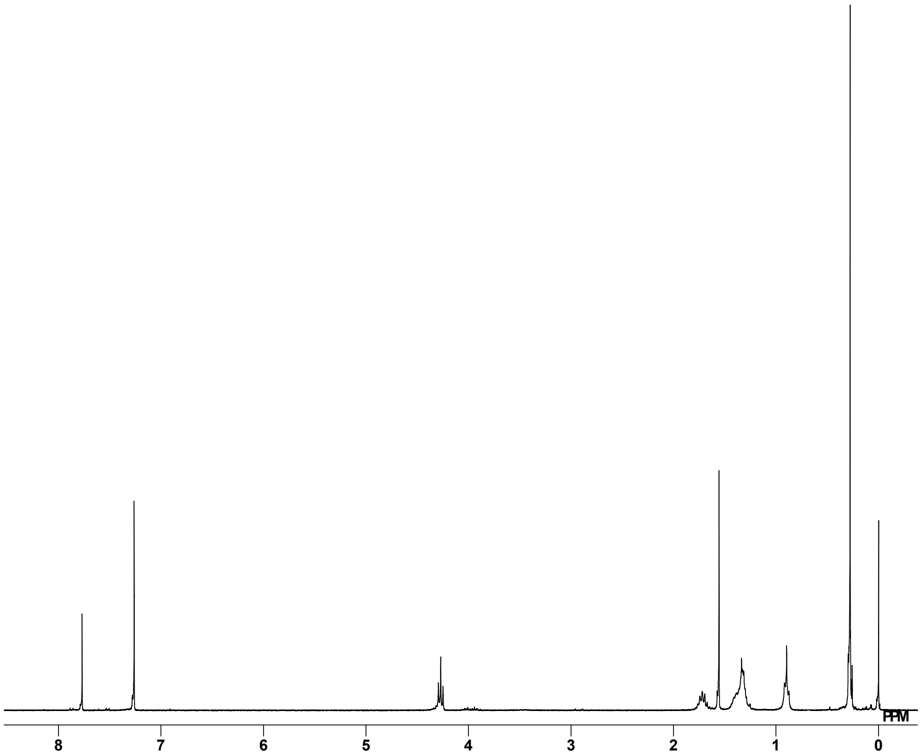

Figure S4 ^{1}H NMR of **4** in $CDCl_3$.

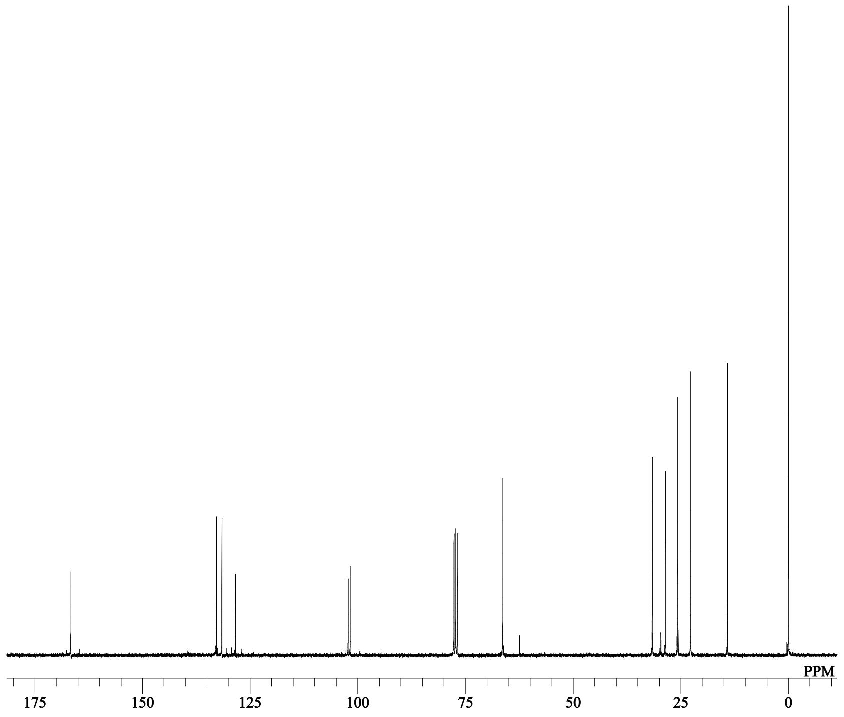

Figure S5 ^{13}C NMR of **4** in $CDCl_3$.

Synthesis of 5. In a 100 mL flask, compound **4** (97.5 mg, 0.185 mmol) was dissolved in THF (30 mL). Then, (n-$C_4H_9)_4NF$ (1 M THF solution, 0.1 mL, 0.1 mmol) was added and the mixture was stirred at room temperature for 1 h. After the addition of water, the organic layer was extracted with CH_2Cl_2, dried over Na_2SO_4, filtered, and the solvents were evaporated. Column chromatography afforded dihexyl 4,5-diethynylphthalate which was directly used for the next reaction. Dihexyl 4,5-diethynylphthalate was dissolved in CH_2Cl_2 (100 mL), and CuCl (30 mg, 0.30 mmol) and *N*,*N*,*N*',*N*'-tetramethylethylenediamine (0.80 mL, 5.4 mmol) were added and the mixture was stirred at room temperature for 12 h. After the mixture was washed with 0.5 M HCl solution, the organic layer was extracted with CH_2Cl_2 and dried over Na_2SO_4. Column chromatography afforded the desired compound as a yellow solid (8.9 mg, 12.7%). For safe storage, this compound was dissolved in CH_2Cl_2 and placed in a refrigerator. ^1H NMR ($CDCl_3$, 300 MHz, 297 K) δ: 7.29 (4H, s), 4.25 (8H, t, J = 6.8 Hz), 1.74-1.65 (8H, m), 1.32-1.31 (24H, m), 0.89 ppm (12H, t, J = 6.8 Hz). ^{13}C NMR ($CDCl_3$, 75 MHz, 297 K) δ: 165.75, 133.25, 133.09, 128.82, 91.44, 86.35, 66.36, 31.39, 28.36, 25.53, 22.50, 13.99 ppm. FT-IR (neat): ν = 2067 (C≡C), 1730 (C=O) cm^{-1}. MALDI-TOF MS (dithranol): $[M+Na]^+$ = 783.99.

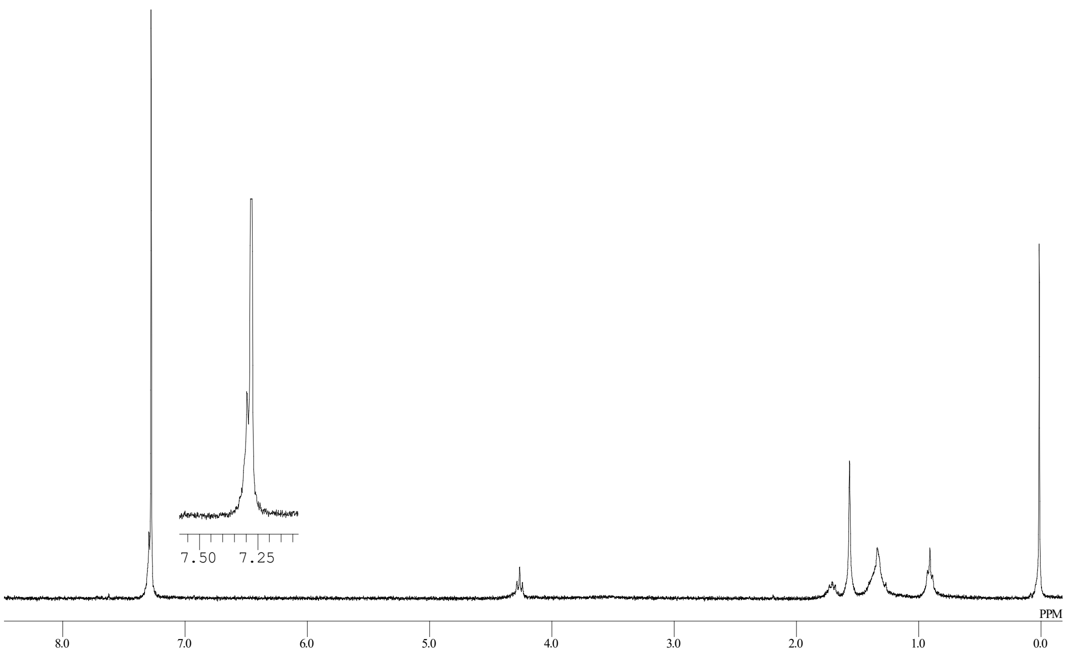

Figure S6 ^{1}H NMR of **5** in $CDCl_3$.

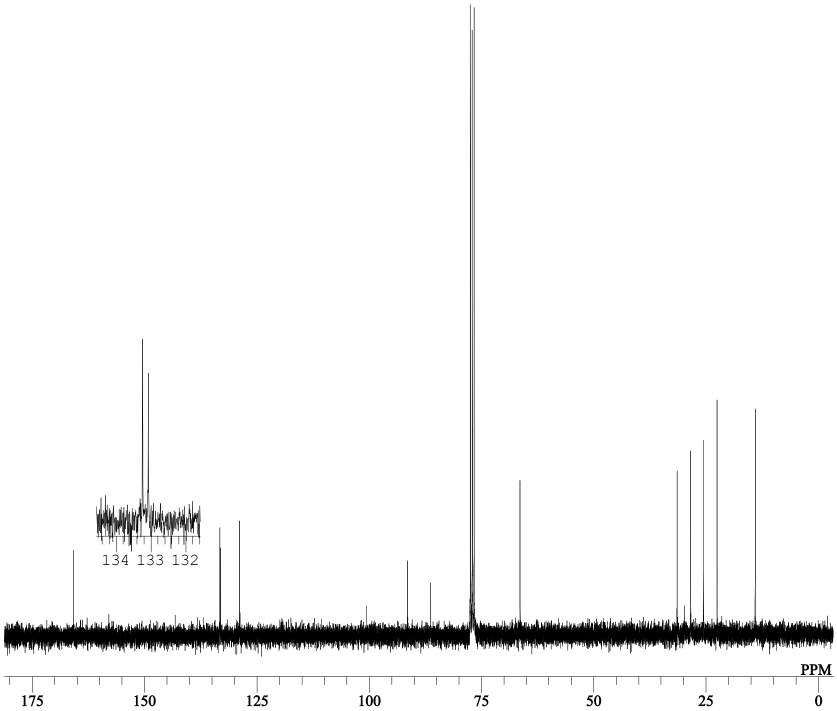

Figure S7 ^{13}C NMR of **5** in $CDCl_3$.

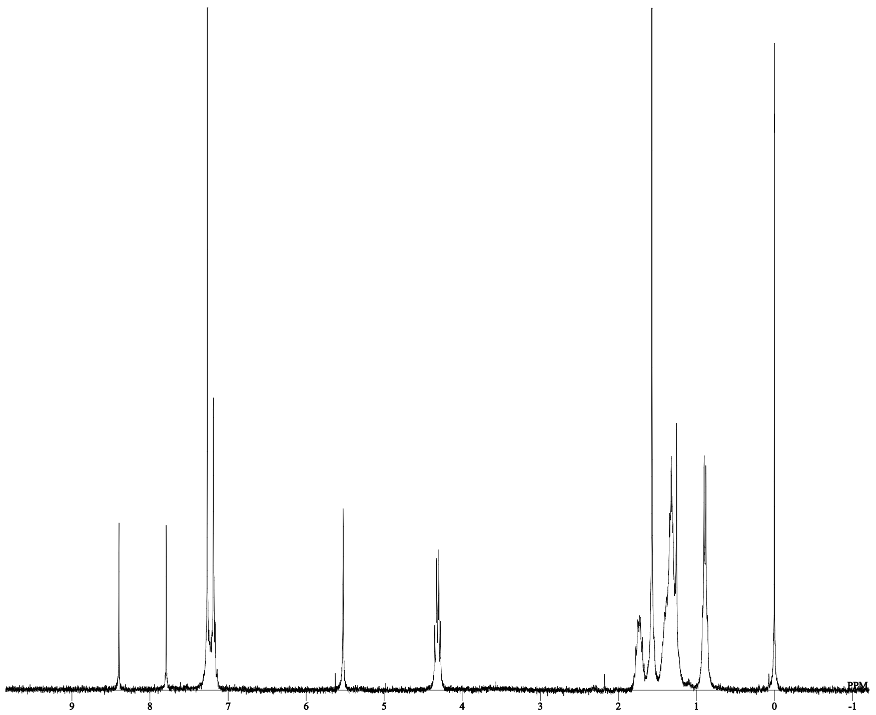

Figure S8 ^{1}H NMR spectrum of **6** in $CDCl_3$.

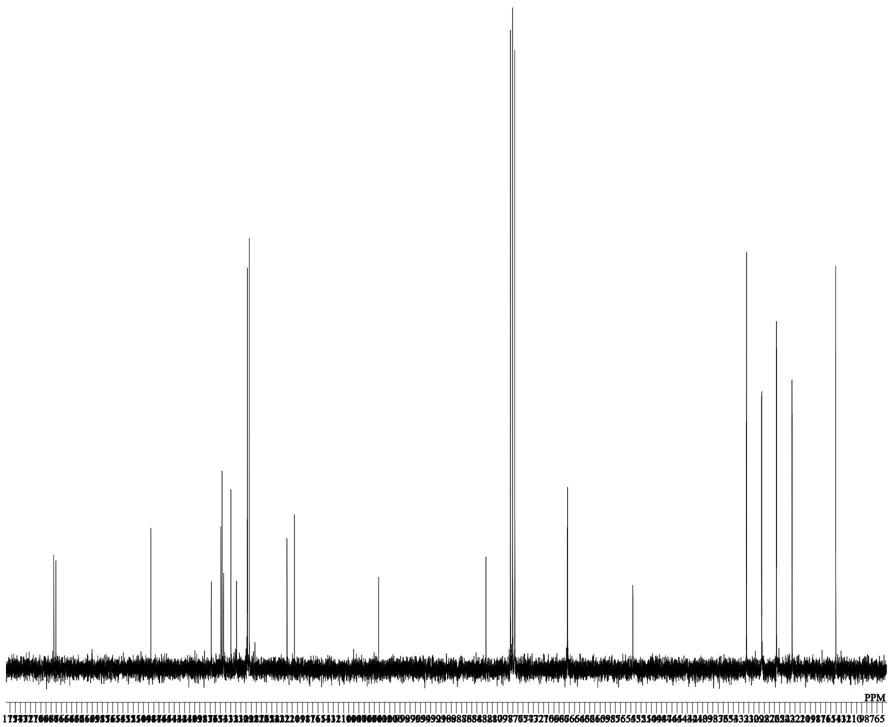

Figure S9 ^{13}C NMR spectrum of **6** in $CDCl_3$.

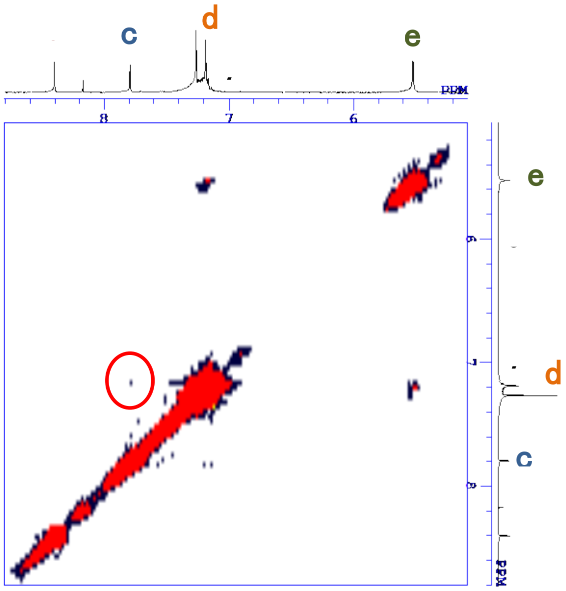

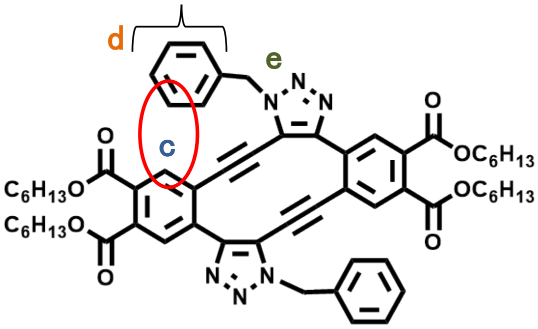

Figure S10 NOESY spectrum of **6** in $CDCl_3$.

3. Kinetic study

The rate constant (k) of the reaction between the DBA and benzyl azide was determined by temperature-dependent ^{1}H NMR measurements using the following equation:

$$\frac{1}{[A]_0 - [B]_0} \ln\left(\frac{[B]_0[A]}{[A]_0[B]}\right) = kt \qquad \text{(eq. S1)}$$

where $[A]_0$ and $[B]_0$ are the initial concentrations of benzyl azide and DBA, respectively, and [A] and [B] are the concentrations of benzyl azide and DBA, respectively, at time t. The detailed conditions are referred to previous report [S2].

Table S1 Activation energy (E_a) of the reaction between DBA and benzyl azide in various solvents

DBA	solvent	E_a (kJ mol^{-1})	Reference
DBA-OHex	DMSO-d_6	93.3	[S2]
DBA-OHex	C_6D_6	86.8	[S2]
DBA-OHex	$CDCl_3$	71.1	this study
**5**	$CDCl_3$	60.9	this study

4. Optical properties

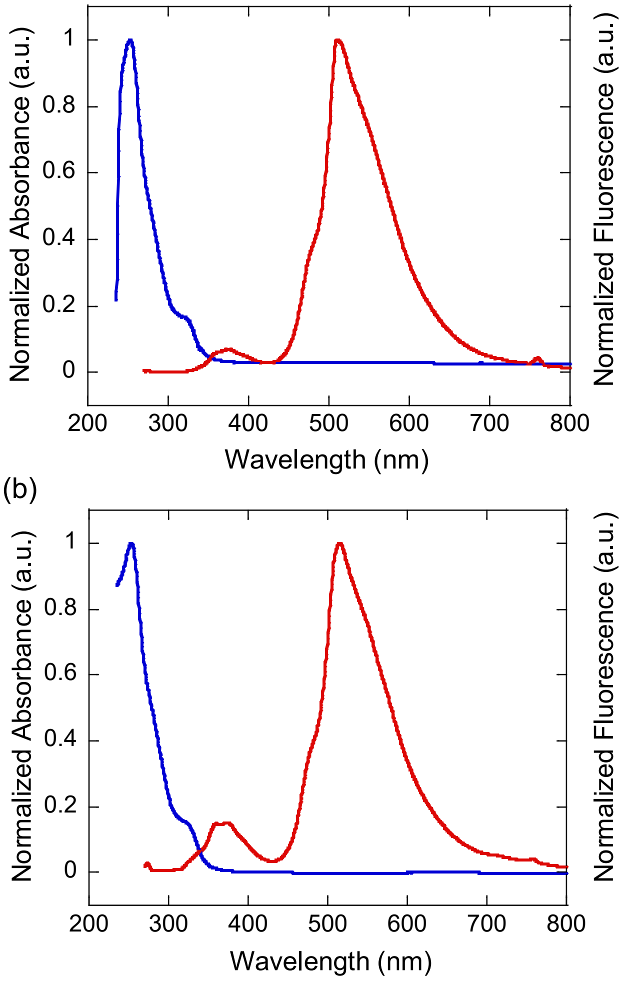

Figure S11 Absorption (blue) and fluorescence (red) spectra of **6a** in (a) $CHCl_3$ and (b) THF.

References

[S1] J. Jiang, B. R. Kaafarani, D. C. Neckers, 'Design, Synthesis, and Properties of New Derivatives of Pentacene', *J. Org. Chem.* **2006**, *71*, 2155–2158.

[S2] S. Fukushima, M. Ashizawa, S. Kawauchi, T. Michinobu, 'Strain-Promoted Double Azide Addition to Octadehydrodibenzo[12]annulene Derivatives', *Helv. Chim. Acta* **2019**, *102*, e1900016.
